# Proteomic profile determination of autosomal aneuploidies by mass spectrometry on amniotic fluids

**DOI:** 10.1186/1477-5956-6-1

**Published:** 2008-01-11

**Authors:** Alain Mange, Caroline Desmetz, Virginie Bellet, Nicolas Molinari, Thierry Maudelonde, Jerome Solassol

**Affiliations:** 1 University of Montpellier I, Montpellier, France; 2 CHU Montpellier, Hôpital Arnaud de Villeneuve, Department of Cellular Biology, Montpellier, France; 3 CRLC Val d'Aurelle, Department of Clinical Oncoproteomic, Montpellier, France; 4 IURC, Department of Biostatistic, Epidemiology and Clinical Research, Montpellier, France

## Abstract

**Background:**

Prenatal diagnosis of chromosomal abnormalities by cytogenetic analysis is time-consuming, expensive, and requires highly qualified technicians. Rapid diagnosis of aneuploidies followed by reassurance of women with normal results can be performed by molecular analysis of uncultured foetal cells. In the present study, we developed a proteomic fingerprinting approach coupled with a statistical classification method to improve diagnosis of aneuploidies, including trisomies 13, 18, and 21, in amniotic fluid samples.

**Results:**

The proteomic spectra obtained from 52 pregnant women were compiled, normalized, and mass peaks with mass-to-charge ratios between 2.5 and 50 kDa identified. Peak information was combined together and analysed using univariate statistics. Among the 208 expressed protein peaks, 40 differed significantly between aneuploid and non aneuploid samples, with AUC diagnostic values ranging from 0.71 to 0.91. Hierarchical clustering, principal component analysis and support vector machine (SVM) analysis were performed. Two class predictor models were defined from the training set, which resulted in a prediction accuracy of 92.3% and 96.43%, respectively. Using an external and independent validation set, diagnostic accuracies were maintained at 87.5% and 91.67%, respectively.

**Conclusion:**

This pilot study demonstrates the potential interest of protein expression signature in the identification of new potential biological markers that might be helpful for the rapid clinical management of high-risk pregnancies.

## Background

Trisomies are the largest group of chromosome abnormalities. In particular, trisomies for chromosome 13 (Patau's syndrome), 18 (Edward's syndrome), or 21 (Down's syndrome) constitute an important indication for prenatal diagnosis, being the three autosomal trisomies found with significant frequency among live births.

For more than 25 years, the prenatal diagnosis of chromosomal abnormalities, based on full karyotyping of fetal cells from amniotic fluid (AF), has been the gold standard. However, *in vitro *culture requires sufficient cells, great technical expertise and time consuming manual procedures. Its main limit is the significant delay in providing the diagnosis (about 10–20 days), which leads to an inevitable and important anxiety period for the parents [1].

More recently, several methods based on fluorescence *in situ *hybridisation (FISH) or semi-quantitative fluorescent PCR (QF-PCR) of short tandem repeats (STRs) have been developed for rapid detection of aneuploidies in high-risk pregnancies, allowing a prenatal diagnosis of chromosomal abnormalities within 24–48 h. Introduced in the late 1980s, FISH analysis of uncultured interphase fetal cells with commercially available multicolour specific probes is presently routinely performed [2]. Although fast and reliable, the prenatal detection of aneuploidies by FISH remains expensive and its use is mainly restricted to pregnancies with anomalies detected by ultrasonography. The use of QF-PCR of STR for fast detection of aneuploidies has been applied to trisomy 21 [3]. Since 1993, this molecular approach has been extensively used by several groups on a research basis [4-9] and the diagnosis of aneuploidies by QF-PCR of STR has now been validated as a reliable method applicable in laboratories [10,11]. However, replacement of full karyotypes with rapid testing for trisomies 13, 18, and 21 by PCR or FISH should result in substantial numbers of liveborn children with hitherto preventable mental or physical handicaps [12].

Amniotic fluid is a significant contributor to fetal health and constitutes a potential rich source of biomarkers for diagnosis of fetal disorders [13]. Particularly, genetic abnormalities such as trisomies may induce alterations of protein expression and/or content which may be useful for diagnosis of many pathologies. Proteomic studies of AF have recently been successfully used to detect premature rupture of membranes [14,15], to diagnose intra-amniotic infection [16-20] and Down syndrome [21-23].

To identify useful and relevant biomarkers for fetal aneuploidies in AF, we used a method based on initial protein fractionation by retenate chromatography on protein chip arrays followed by SELDI-TOF/MS. We examined aneuploidy-specific protein signatures in amniotic fluid of pregnancies at the 17^th ^week of gestation with trisomies 21, 18 and 13 foetuses, compared to chromosomally normal ones using 3 chromatographic conditions.

## Results

### Reproducibility

In order to evaluate the variability of SELDI, we first tested the experimental reproducibility using a pool of normal AF, spotted randomly onto each array together with the tested AF samples. The peaks generated for reproducibility purposes were obtained using the CM10, Q10, H50 and IMAC30-Cu chips. The coefficients of variation of 15 reliable peaks were calculated by average peak intensity values derived from 20 different runs. Acceptable average CVs of 10 and 17% were obtained for intra and inter-assay variability, respectively, which is consistent with previously reported studies of SELDI [24,25].

### Representative biomarkers: Univariate analysis

**Figure 1:**
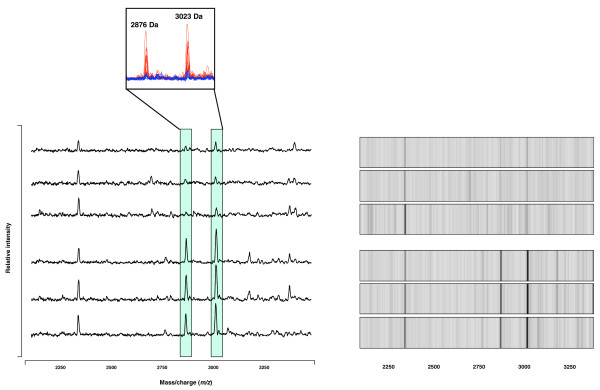
Representative SELDI-TOF spectra (left panel) and gel (right panel) views of selected biomarkers obtained from normal and aneuploid AF samples. For both protein peaks, the data of three normal karyotypes samples (N, upper panel) and three aneuploidy karyotypes samples (A, lower panel) are shown. Frames indicate the positions of two peaks at 2876 and 3023 Da, respectively, both overexpressed in aneuploid samples. Overlay of protein mass spectra is represented in the upper box. Protein mass spectra obtained from aneuploid (red) and normal (blue) AF are superimposed.

Twenty-eight AF samples corresponding to the training set (15 aneuploidie and 13 normal samples) were analysed using four different array surfaces. Trisomy 21 was diagnosed by standard cytogenetic methods in 9 cases, trisomy 18 in 4 cases, and trisomy 13 in 2 cases. Using the ProteinChip and Biomarkers Wizard software, a total of 208 protein peak clusters in a range of 2.5 to 50 kDa were obtained from the training set: 52 with the CM10, 79 with the Q10 and 77 with the IMAC30-Cu arrays. The H50 array was not subjected to analysis due to the weak number of peaks detected in preliminary experiments. According to our first statistical criteria, we selected 40 (11, 16 and 13 for CM10, Q10 and IMAC30-Cu, respectively) of 208 protein peaks that were significantly differentially expressed between aneuploidie AF and control samples (*p *< 0.05). Among these 40 peaks, 12 of them were overexpressed and 28 of them underexpressed in aneuploidie AF as compared to the control samples. Diagnostic values of each of these 40 differentially expressed peaks were high, with an AUC of the receiver operator characteristic curve (AUC-ROC) ranging from 0.71 to 0.91 according to the different protein peaks. A representative example of 3 discriminating protein peaks are shown in Figure 1 and the mean intensity, the p-value and the AUC value of the 40 peaks were given in Table 1.

**Table 1:** Forty statistically significant protein peaks are differentially expressed in normal and aneuploid AF samples

				**Peak intensity ^(d)^**		
					
***m/z *^(a)^**	**Surface array**	***p*-value ^(b)^**	**AUC ^(c)^**	**Normal**	**Aneuploidies**	**Fold Change**
6080	CM10	0.00001	0.918	1.66	9.50	5.7
2876	CM10	0.0006	0.861	6.37	20.96	3.3
15944	IMAC	0.0013	0.838	4.12	1.99	0.5
3023	CM10	0.0015	0.837	10.06	27.88	2.8
7970	IMAC	0.0016	0.833	4.78	2.67	0.6
6194	CM10	0.003	0.817	1.55	9.75	6.3
3903	IMAC	0.0037	0.810	7.85	2.90	0.4
8455	CM10	0.0065	0.793	38.28	26.92	0.7
22214	CM10	0.0065	0.793	0.30	0.16	0.5
9626	IMAC	0.0068	0.790	5.19	3.01	0.6
24720	Q10	0.0079	0.781	0.58	0.48	0.8
9467	IMAC	0.0105	0.776	6.05	4.02	0.7
3173	Q10	0.0118	0.768	4.64	2.04	0.4
18977	IMAC	0.012	0.771	0.61	0.29	0.5
4323	Q10	0.0134	0.763	5.66	9.47	1.7
5655	Q10	0.0134	0.763	5.47	2.92	0.5
14017	IMAC	0.0137	0.767	4.82	2.65	0.5
3184	CM10	0.0151	0.764	2.93	8.41	2.9
3435	IMAC	0.0157	0.762	2.66	9.04	3.4
8660	CM10	0.0172	0.760	4.95	3.00	0.6
9276	IMAC	0.0178	0.757	1.90	4.11	2.2
12289	IMAC	0.0178	0.757	4.97	3.33	0.7
15056	Q10	0.0194	0.750	0.78	1.31	1.7
3383	CM10	0.0196	0.755	7.24	12.71	1.8
13761	IMAC	0.0202	0.752	6.15	4.10	0.7
7655	Q10	0.0245	0.741	4.52	6.56	1.5
14014	Q10	0.0245	0.741	4.56	2.72	0.6
11670	CM10	0.0251	0.744	3.19	1.92	0.6
4954	IMAC	0.0259	0.743	14.73	10.19	0.7
7007	Q10	0.0275	0.737	3.85	1.98	0.5
6892	Q10	0.0308	0.732	4.34	3.26	0.8
8203	Q10	0.0308	0.732	5.80	3.45	0.6
9159	Q10	0.0308	0.732	9.03	7.82	0.9
11314	Q10	0.0344	0.728	6.90	4.10	0.6
8574	IMAC	0.0367	0.729	12.48	7.55	0.6
4519	Q10	0.0472	0.714	1.44	2.27	1.6
4901	Q10	0.0472	0.714	3.61	2.25	0.6
13809	Q10	0.0472	0.714	4.20	2.73	0.6
49297	Q10	0.0472	0.714	0.21	0.16	0.8
11303	CM10	0.0499	0.716	2.62	1.90	0.7

*a) m/z*: mass/charge ratio of the protein peak.

*b) P-values *were calculated using parametric Mann-Whitney test.

c) AUC (area under the ROC curve) were calculated using normal amniotic fluids as reference.

d) Averages of peak intensities.

### Representative Biomarkers: Multivariate Analysis

**Figure 2:**
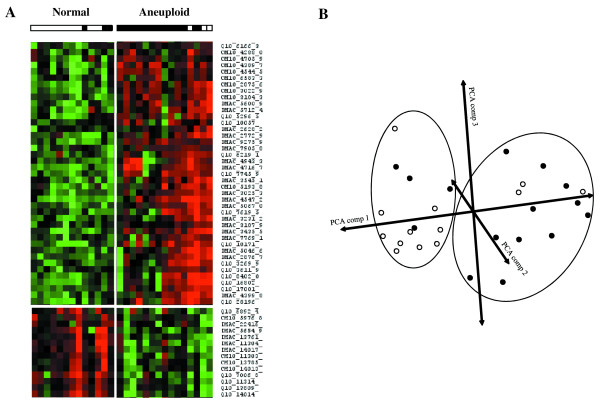
Heat cluster map and principle component analysis (PCA) of aneuploidie amniotic fluid and normal samples. All 208 protein peaks from 2.5 to 50 kDa mass range and 3 different surface arrays were pooled to generate a heat cluster map (A) and PCA diagram (B). The PCA diagram illustrates the segregated samples based on normal (white circles) and aneuploidie (black circles) samples.

A heat cluster map, indicating up and down regulation of possible biomarker candidates, was generated for a multivariate analysis of the samples in a training set. The heat cluster map generated represents all the possible biomarker peaks between 2.5 and 50 kDa, from all three protein chips, in which differences in expression were observed between normal and aneuploid amniotic fluids. On the basis of the colour pattern (red indicating up regulation and green indicating down regulation) generated, it is clear that differences exist between normal samples compared to the aneuploid samples (Figure 2A). Upon closer observation of the heat cluster map, it is apparent that 3 aneuploid samples (2 trisomies for chromosome 18 and one for chromosome 21) were misclustered and have more possible biomarkers in common with the normal samples as compared to the aneuploid samples. In addition to the heat cluster map, a second multivariate analysis was generated using a PCA diagram. The PCA allowed us to visualize the relationship between the normal and aneuploid samples in a three-dimensional view based on all the potential biomarkers identified. Blue circles represent normal samples in contrast to the red circles, which represent aneuploid samples. Based on the PCA diagram, we could conclude that the same 3 amniotic and 3 normal samples are still misclassified (Figure 2B). Markers chosen as representatives had a *p *< 0.05 indicating their significance.

To construct a multivariate classifier for aneuploidies, we used the 208 protein peaks obtained by SELDI analysis to train a SVM classification algorithm taking into account biomarker inter-correlations. SVM is a supervised learning technique that constructs an optimal separating hyperplane from the training set with an aim of classifying the test set [26-28]. It achieved an overall classification accuracy of 92.86%, sensitivity of 93.33%, and specificity of 92.31% in distinguishing normal samples from aneuploid samples. To construct a multivariate classifier, we also performed a preselection step to allow an optimal identification of differentially expressed peaks. Logistic regression was used to construct a multiprotein classifier composed of two protein peaks (CM10, 6194 kDa- and Q10, 3173 kDa). The composite index yielded higher AUC value (0.928) than the individual protein peaks (0.817 and 0.768). Table 2 indicates the mean performance of both classification methods. In order to validate our classifiers, we next performed an independent validation test on 24 AF samples (12 normal and 12 aneuploid samples). SVM algorithm was used to classify the test set. Trisomy 21 was diagnosed by standard cytogenetic methods in 8 cases, trisomy 18 in 3 cases, and trisomy 13 in 1 case. Results indicated that overall classification accuracies were maintained in the validation phase with 87.5% (sensitivity 83.33%, specificity 83.33%) and 91.67% (sensitivity 83.33%, specificity 100%) for SVM classification and logistic regression indexes, respectively. Considering an estimated prevalence of aneuploidies in the general population of 1/800 [29], we evaluated the PPV and the NPV for both classifiers (Table 2).

**Table 2:** Diagnostic performance of classifiers

				**Tested population**		**General population**	
					
**Model**	**Accuracy**	**Sensitivity**	**Specificity**	**PPV**	**NPV**	**PPV**	**NPV**
**SVM multiprotein index**							
**Learning population**	92.86	93.33	92.31	93.33	92.31	1.50	99.99
**Testing population**	87.50	83.33	83.33	83.33	83.33	0.62	99.97
**Logistic regression multiprotein index**							
**Learning population**	96.43	93.33	100.00	100.00	92.86	100.00	99.99
**Testing population**	91.67	83.33	100.00	100.00	87.71	100.00	99.98

Positive Predictive Value (PPV) and Negative Predictive Value (NPV) were estimated for the tested population and for the general population (with a prevalence of 1/800).

## Discussion

With the development of obstetric ultrasound examinations for prenatal care, fetal anomalies are often found without the objective of diagnosis [30]. These findings do not allow identification of a specific chromosome region as a target for prenatal diagnosis. Although microarray analyses have been used to identify many molecular events and to analyse variations in gene expression associated with aneuploidies [31-33], the level of mRNA expression does not systematically reflect the proteome in a cell. Indeed, translation of mRNA into proteins depends on several post-transcriptional and post-translational events (glycosylation, phosphorylation, acetylation, etc.). Hence, direct measurement of protein expression may determine cell dysfunction more effectively. Proteome analysis is based on the combination of three technologies: a resolutive method for separating proteins as a function of different physico-chemical criteria (mass, pI, hydrophobicity, etc.), mass spectrometry (MS) and bioinformatic tools. It is therefore possible to identify proteins from MS data and quantitatively analyse their expression simultaneously. Bi-dimensional gel electrophoresis coupled with MALDI-TOF MS (Matrix Assisted Laser Desorption Ionization – Time of Flight) remains the most powerful resolutive method to separate complex mixtures of proteins and to compare variations of protein expression in a pair of samples, such as normal versus tumour. The proteins of interest are identified by their peptide mass fingerprints, obtained after trypsin digestion in gel. This approach has been used in several studies since 2004 to identify potential biomarkers in amniotic samples for detection of premature rupture of membranes [14] and trisomy 21 [22,34]. Although bi-dimensional gel electrophoresis has proved effective in many fields, its use in clinical proteomics is limited. Indeed, it has a labour-intensive and time-consuming nature rendering analysis of more than ten samples impossible. Therefore, it is only of limited value at the condition when tested individuals may elicit significant biological variations, such as those in multiple normal tissues from the same organ obtained from a group of normal individuals or those in different tumours of the same type of cancer but derived from different patients.

Recently, progresses in nanotechnologies based on liquid or surface-affinity chromatography have generated new broadband proteomic analysis techniques for application to diagnosis of human diseases. SELDI-TOF combines surface-affinity chromatography and mass spectrometry. It separates and detects peptide and protein peaks from various biological samples such as amniotic fluid, LCR, serum or tissue. Using the automated clinical proteomic method, hundreds of samples can be analysed within a short period of time allowing statistical analyses to detect significant differences between two groups of samples beyond the presence of individuals' biological variations [35]. SELDI-TOF has been previously used to detect aneuploidies in amniotic fluid [21,23]. Bush *et al*. described a clustering algorithm based on protein profiles obtained from H50 and SAX2 chip surfaces. Wang *et al *proposed a two-step proteomic analysis model based on a C18 purification set followed by a weak cation exchange. Both studies presented their classification method but did not clearly indicate discriminatory protein peak molecular weights, rendering an independent validation study not realizable. In our study, we have identified, using SELDI-TOF, the MS-generated proteomics profiles from 52 uncultured amniotic fluids to be able to distinguish normal foetuses from those with abnormal karyotypes. We found in an univariate analysis 40 peaks as potential biomarkers, of which 12 were overexpressed and 28 underexpressed, in aneuploid AF as compared to the control samples. All were considered statistically significant (*p *< 0.05) and AUC values were correct, proving the validity of their accuracy. The most important steps in proteomic studies were the differential and statistical analysis of all data from the spectra. To analyse our data, we searched for a combination of methods that could reliably detect aneuploidies in a high-risk group of women. Since there is no "gold-standard" method for classification of mass spectrometry data, we were interested in testing the validity and robustness of our methods. Using a SVM classification algorithm, we observed a classification accuracy of 92.9% with a sensitivity of 93.3%, and specificity of 92.3%. Since one of the challenges of SELDI data is to reduce the high-dimension of the spectra, we also extracted combination of markers from all the 208 protein peaks by logistic regression. This analysis identified combination of two protein peaks with an AUC of 0.928, a classification accuracy of 96.4%, a sensitivity of 93.3% and a specificity of 100%. Importantly, these values remained high in an independent validation set proving the robustness of these methods. Considering the sensitivity and specificity of our classifiers and the expected prevalence of aneuploidies in the general population, one can expect an extrapolated PPV of 0.4% and a NPV of 99.99% for the SVM classifier index, and a PPV of 100% and a NPV of 99.98% for the logistic regression classifier index. Thus, our proteomic classifiers could be more particularly used to rapidly screen, within two hours, for aneuploidies in a high-risk population in whom amniocentesis is indicated and when the diagnosis is urgently need. Due to the relatively high sensitivity and the perfect NPV of the test, we minimized the risk of missing an affected pregnancy. However, clinical trials with more samples are needed to verify the interest of this rapid detection by proteomic analysis.

The discriminating proteomic profiling model used in this study may show some limits. In particular, some aneuploid samples were misclassified (two trisomies 18 and one trisomy 21). The discrepancy between the cytogenetic and the molecular results may be explained by the fact that we analysed AF samples derived from various abnormal karyotypes as a group. In addition, we cannot exclude some sample contaminations with maternal cells. It would be interesting to discard all amniotic fluids suspected of blood cell contamination (determined by microscopic examination for red blood cells). This should allow the reallocation of some of these individuals into the correct group. Other limitation is that we analyzed AF samples from different aneuploidies as a group. Then, whether we are unable to specify trisomy 21, 18 or 13 markers individually, we can detect rapidly various types of aneuploidies in one experiment, extending the potential of clinical application. Increasing number of samples in further studies should allow to individualise markers for specific trisomies detection.

We are convinced that, for the moment, none of the different proteomic approaches will replace chromosome analysis and karyotyping on cultured amniocytes [12]. However, a major drawback of this procedure is the necessity for viable cells to culture for prenatal diagnosis, which may take up to 2 weeks. Prenatal diagnosis based on amplification of STR detection and amplification on uncultured amniotic cells has proved reliable, avoiding the need to culture cells and reducing the time required for a diagnosis. However, this technique is time-consuming and requires intact cells. Our proteomic assay was designed for a rapid detection of common trisomies of chromosome 13, 18 and 21, focusing on high-risk pregnancies with anomalies detected by ultrasonography, as an alternative to FISH or PCR quantitation assay. Our results should be considered as screening or preliminary tests of aneuploidies in high-risk pregnancies that should be confirmed by cytogenetic analysis. In addition, this proteomic method allows further research to be conducted in this area, in particular, in serum samples of women with normal and aneuploidy fetuses for non-invasive prenatal diagnosis.

## Conclusion

In conclusion, this report describes a proteomic approach combining ProteinChip technology and rule-based analysis that allow a screening for fetal aneuploidies with good accuracies. The assay could be used as an adjunct for traditional cytogenetic analysis as proposed by PCR quantitation array or FISH analysis. This preliminary study supports the view that SELDI-TOF is a valuable tool to identify biomarkers for aneuploidies diseases in amniotic fluid but also in serum.

## Methods

### Supernatant specimens of amniotic fluid

A total of 52 pregnant women underwent amniocentesis for prenatal chromosome analysis between gestational weeks 14 and 17. These women were referred to the prenatal diagnosis unit of the university hospitals of Clermont-Ferrand and Montpellier. The referral criteria were maternal age (38 years which is the French cut-off age for free-of-charge karyotyping), positive biochemical screening for Down syndrome, abnormal fetal ultrasonic scan, positive family history of chromosomal rearrangement, or parental anxiety. Ethical approval for the study was obtained under the assumption that results were not reported to the referring clinicians. Through the transabdominal approach, about 20 ml of AF was tapped from each patient. Conventional chromosome analyses were carried out for each sample. Two ml of AF were used for the proteomic study. Amniotic cells were collected by centrifugation at 1500 rpm for 5 min. After removing the supernatant, cells are frozen at -80°C until analysis. Before proteomic analysis, cells were lysed in 50 mM Tris pH 7.5, 100 mM NaCl, 5 mM EDTA, 0.5% Triton X100 and 0.5% CHAPS. Protein concentration was measured using the EZQ Protein quantitation kit (Molecular Probes) according to the manufacturer's protocol. From the 52 samples, training and testing populations were randomly done.

### Proteomic analysis

Each AF samples were randomly applied on four different ProteinChip array surfaces (Ciphergen Biosystem, Inc) in a 96-well format: hydrophobic (H50), cation-exchange (CM10), anion-exchange (Q10) and metal binding (IMAC30-Cu). IMAC arrays were pre-treated twice with 50 μL 100 mM CuSO4 on a shaker for 5 min, followed by two washes with 100 μL water. ProteinChip arrays were equilibrated twice with 100 μL binding buffer on a shaker for 5 min and 50 μL binding buffer was added to the surface spots. Five μg of each sample was added to the binding buffer and the arrays were incubated for 30 min on a shaker. Arrays were washed three times with 100 μL binding buffer, followed by a final water wash. For the H50 arrays the final wash was in binding buffer. Arrays were removed from the bioprocessor, allowed to air dry and 1 μL of 50% SPA was applied twice to the spots. Binding buffers used for the different arrays were 10% CH3CN, 0.1% TFA for H50; 100 mM NaAC pH 4.0 for CM10; 100 mM Tris-HCl pH 9.0 for Q10; and 100 mM phosphate buffer pH 7.0, 500 mM NaCl for IMAC30-Cu. Finally, arrays were analysed on a PBS-IIc ProteinChip Reader. Data were averaged over 200 transients for each spot and were performed in the range of 2.5 to 50 kDa. Training and testing phases were run in independent experiments.

### Reproducibility

Mass detection accuracy of PBSIIc was calibrated externally by using the All-in-1 protein II molecular mass standards (Ciphergen Biosystems). Reproducibility was estimated using a control sample which was randomly spotted on each chip to measure the variability of fractionation, on-chip spotting, and data acquisition. Intensity values for the selected peaks were used to calculate coefficients of variation (CV). The reproducibility analysis was performed for all surface conditions and spectra were collected following the experimental protocols described above.

### Peak detection

Analysis of peaks was performed using the ProteinChip and Biomarker Wizard software from Ciphergen Biosystem. Spectra were background subtracted and the peak intensities were normalized to the total ion current of m/z between 2.5 and 50 kDa. Automatic peak detection was performed with the following settings: i) signal-to-noise ratio at 4 for the first pass and 2 for the second pass, ii) minimal peak threshold at 15% of all spectra, iii) cluster mass window at 0.5% of mass. The resulting CSV file containing absolute intensity and m/z ratio was exported into Microsoft Excel (Microsoft, Redmond) for subsequent analysis.

### Statistical data analysis

Univariate statistical analysis of SELDI-TOF peak masses and relative intensity values was conducted with the nonparametric Mann-Whitney U-test. Differentially expressed proteins were defined with a *p*-value < 0.05. Peak intensity data were then preprocessed before multivariate statistical analysis using logarithmic transformation. Data were analysed using various statistical algorithms such as hierarchical clustering, principal component analysis (PCA) and support vector machine (SVM) analysis, implemented in the MultiExperiment Viewer software (Mev, version 4) [36]. Hierarchical clustering and PCA were not used as predictive tools, but purely as a mean to understand and visualize the data structures being analysed. All m/z values were subject to average-linkage hierarchical clustering, using Pearson correlation as a distance metric. The peaks and samples were organized by creating dendrograms. We also performed dimension reduction using PCA, which converted higher-dimensional space spanned by differential peaks into less-redundant and lower-dimensional space of several principal components. A class prediction model was defined from the training samples using SVM. SVM was chosen because this learning scheme was known to perform well in the challenging situation in which the number of samples in the data set is not large compared with the number of attributes. In our case, the attributes were the peaks. SVM found the maximum margin hyperplane, which was the hyperplane separating the two classes of samples in a n-dimensional space while maximizing the distance between the hyperplane and the closest training point. On the other hand, logistic regression was used for peak selection and to construct a multiprotein classifier from all the 208 protein peaks. To maximize the use of our relatively small data set, we have assessed the performance of our class prediction models with an external validation scheme. The classifier was thus used to predict the class validation samples.

## Competing interests

The author(s) declare that they have no competing interests.

## Authors' contributions

AM participated to the design of the experiment, performed the proteomic analysis and drafted the manuscript.

CD and VB carried out the SELDI-TOF analysis.

AM and NM carried out the statistical analysis.

TM was the head of the laboratory

JS coordinated the study and wrote the final version of the manuscript.

All authors read and approved the final manuscript.
